# Re-imaging malaria in the Philippines: how photovoice can help to re-imagine malaria

**DOI:** 10.1186/s12936-015-0770-8

**Published:** 2015-06-24

**Authors:** Dalia Iskander

**Affiliations:** Department of Anthropology, Durham University, Dawson Building, South Road, Durham, DH1 3LE UK

**Keywords:** Re-imagining malaria, Philippines, Photovoice

## Abstract

**Background:**

This paper responds to a recent call for malaria to be re-imagined by: explaining why it needs to be re-imagined; offering one possible way in which this can be done; and describing some of benefits for malaria control when it is.

**Methods:**

This study involved conducting a 15-week photovoice project with 44 predominantly ethnically Palawan school-going children in the municipality of Bataraza in the Philippines. The primary aim was to critically examine how facilitating children to take their own pictures of malaria could alter their understanding of it as well as the practices that they then engaged into prevent and treat it.

**Results and discussion:**

During the photovoice process, participants responded to the question, ‘what does malaria mean to you?’ by photographing multiple versions of malaria. Some of these versions align with biomedical conceptions and mirror common images of: its sources (e.g. mosquitoes); symptoms (e.g. fever); prevention practices (e.g. use of mosquito nets); diagnostic practices (e.g. use Rapid Diagnostic Tests) and treatment practices (e.g. use of anti-malarial drugs). However, in addition to these depictions, participants also took images of malaria that aligned with more local understanding of the body, health and well-being, which are often neglected by health practitioners. In the case of the Palawan, these versions of malaria are structured around the central tenet of balance. Participants therefore photographed themselves and members of their family and community engaging in a number of practices, which are orientated towards restoring and maintaining balance. As well being an effective means to illuminate multiple malarias and the practices that surround them, photovoice also enabled participants to learn new things and significantly, teach these things to others using their images.

**Conclusion:**

Photovoice is an effective method for re-imaging malaria. It allowed participants to *depict* and *describe* multiple versions of malaria and the practices that they engage in *in context.* Photovoice also had a potentially transformative effect. It acted as a means for participants and researchers to: visually *depict* everyday practices; collectively gain a deeper understanding of this doing; and then seek ways in which to make changes *in line* with this joint understanding.

## Background

At a recent workshop held at the London School of Hygiene and Tropical Medicine, the delegates, composed of social scientists working in malaria research and control at 11 world-wide institutions, recommended that malaria be ‘re-imagined’. This paper responds to this suggestion by: making the case for why malaria needs to be re-imagined; offering one possible way in which this can be done; and describing some of benefits for malaria control when it is.

### Why does malaria need to be re-imagined?

Multiple authors have documented how groups involved in malaria control, including those in medicine, academia, politics and philanthropy, continue to use language which is infused with metaphor to describe both malaria and the attempts to deal with it [1, 2]. In particular, the current discourse around malaria still retains many of the military metaphors that were particularly prevalent in the post-war eradication era [3]. Reference is still made to ‘wars’, ‘battles’ and ‘campaigns’ being ‘fought’ with the use of ‘strategies’, ‘weapons’, ‘tools’ and even ‘silver bullets’, in the attempt to ‘combat’ malaria. However, a combination of the failure of early eradication campaigns and the reduction in malaria cases particularly over the last decade have resulted in changes to the semantic field which now includes more positive constructions, frequently including concepts, such as ‘sustaining the gains, ‘making durable progress’, ‘shrinking the malaria map and ‘maintaining progress’. While the discourse related to malaria may have moved on from purely military symbolism, implicit in all of these depictions is the idea that malaria is a unified ‘enemy’ that can be ‘monitored’, ‘controlled’, ‘eliminated’ or even ‘eradicated’. These approaches are couched in the objectification of malaria as this complex phenomena is reduced to a single, definable ‘disease’. Other versions of malaria that do not align with this hegemonic conception are consequently silenced, ignored and neglected [2]. Furthermore, this singularized malaria is conceived of as being appropriately handled using a *complicated mix* of standardized technical solutions [4].

### How can malaria be re-imagined?

The discourse surrounding disease affects the way in which it understood, experienced and dealt with. One of the strongest ways in which these depictions are created and propagated is through the visual images that surround illness [5]. In the case of malaria, biomedical depictions of the ‘life-cycle’ have become globally dominant and reduce malaria to a *single acultural* entity that is the product of interaction between three isolated components—the parasite, the mosquito vector and the human. Other ‘messier’ cultural, social, infrastructural, economical factors that contribute to the construction of *multiple* versions of malaria in local contexts are thus excluded from diagrams, conceptions and solutions [4]. As a result, universal and standardized technological solutions are brought to the fore and targeted towards one or more of the components of malaria, albeit in complex ways. One potential way to re-cast malaria back into broader terms that takes better account of multiple malarias is to expand the kinds of images that are associated with the disease. These images, rather than presenting one universal ‘biomedical’ depiction of malaria, would reflect its local, social and multiple natures. This process of re-*imaging* would in turn, help to re-*imagine* malaria and broaden the way in which control is both understood and applied *in context*.

The methodology of photovoice presents one viable way of broadening the images available of these multiple malarias, how they are understood and the solutions that are adopted to deal with them. Photovoice (PV) is a method of Participatory Action Research (PAR) that was developed in the mid-1990s by Wang and colleagues [6–9]. It refers to the process in which participants: take photos that document aspects of their lives or social realities; select images to reflect on and discuss, raise emergent issues, themes and theories in group discussion; and communicate findings to the wider community and decision-makers. As well as being a useful method for a community to communicate ideas and conduct an assessment of their needs, the ultimate aim of PV is to promote individual and social change (ibid). According to current literature, PV is effective because of its ability to ‘empower’ individuals to make changes to their lives by making them ‘critically conscious’ (ibid.). By becoming more aware of the their lives and practices, participants are able to develop a clear, mental conceptualization of a different future and then take increased cognitive control over decisions that affect their lives (ibid.).

This study involved conducting a PV project with predominantly ethnically Palawan school-going children in the municipality of Bataraza in the Philippines. The primary aim was to critically examine how facilitating children to take their own pictures of malaria could alter their understanding of it as well as the practices that they then engaged into prevent and treat it. This paper documents how PV is effective in illuminating both the various characters of malaria as well as the multiple ways, often disregarded by health professionals, which are being employed by local people to deal with them in *local* contexts. Significantly, the process of re-imaging malaria had potentially transformative effects. Not only did participants learn new things for themselves, but using their images, were also able to teach others what they had learnt. As a result, they were able to alter both their own and other people’s malaria related-health practices.

## Methods

The study was conducted with participants living in the municipality of Bataraza on the island of Palawan in the Philippines. There has been a 75% reduction in the number of reported cases of malaria in the last decade and the Philippines is now moving towards the goal of eliminating the disease altogether by 2020 [10]. However, the island province of Palawan is known to be one of the more malarious areas in the Philippines and the municipality of Bataraza in Palawan has been identified as an area with particularly high prevalence of malaria.

The primary aim of this study was to evaluate the effect of photovoice in *illuminating* and *altering* malaria-related health practices. In order to do this, a quasi-experimental study design was employed, triangulating qualitative and quantitative data in order to evaluate the effect of photovoice on the malaria-related health practices of young people and their adult caregivers.

Participants were selected from four elementary schools in two *barangays* (smallest political unit) in Bataraza (Inogbong and Bonobono). *Barangays* were selected following discussions with local health staff who gave advice regarding areas which were considered highly endemic (≥one case per 1,000 population) in terms of malaria transmission. Within each *barangay*, two schools were selected and then randomly assigned to control and intervention groups using a coin toss. Schools were selected based on criteria that included: location in *barangays* that had highest rates of malaria in Bataraza in 2012; location in the same *barangay*; similar distance from health services (in Bonobono, both schools are located about 1 km from the *Barangay* Health Station (BHS) and 6 km from the Rural Health Unit (RHU) and in Inogbong, both schools are 1 km from BHS and 7 km from RHU); similar ethnic population; similar ecological setting; community recognition of malaria or febrile illnesses as one of the major problems affecting young people; and school and community willingness to participate in the study. In Inogbong, 25 young people were recruited from grades 3, 4 and 5 in the intervention school (Matyag Elementary School) and 40 from grades 3, 4 and 5 in the control school (Saray Elementary School). In Bonobono, 19 young people were recruited from the intervention school from grades 3 and 4 (Taysay Elementary School) and 24 from grades 3 and 4 in the control school (RVEMS). The total population at baseline consisted of 108 young people.

In addition, one adult from each young person household was invited to participate in the study. In total, 83 adult caregivers were recruited at baseline. In cases where caregivers living in the same household were not able to participate, adults who lived outside the young person’s household but who also contributed to care giving were permitted. Only one caregiver did not live in the same house as the young person. There were fewer caregivers than young people as 16 young people did not have an adult present at recruitment due to illness or work commitments and 14 caregivers had more than one child recruited into the study.

Questionnaires were conducted with both intervention and control groups in order to assess knowledge and practices related to malaria at baseline. Questionnaires were administered to both young people and adult caregivers from control and intervention groups in Tagalog using trained translators.

In the two intervention groups, a 15-week photovoice project was conducted with young people in schools. Pupils were divided into five groups of between seven and ten students in each group and sessions were conducted during lunch breaks in each school. Here, photovoice was the used as a Participatory Action Research (PAR) method in order to collect and interpret qualitative data that *depicts* practices *with* research participants in order for both researchers and participants to collectively gain a deeper understanding of these issues and then potentially alter practices *in line* with this joint understanding. In terms of the photovoice sessions, a day-long training course was conducted for translators and teachers from each school who then acted as co-facilitators in the project. Each group of students met once a week on a regular day for 15 weeks and the project was broadly divided into three phases. Consistent with the literature on photovoice, the aim of the initial sessions was to: introduce the project and methodology; bond the group; and provide basic training of photography and camera use [6–9]. Once young people felt comfortable with the idea of the methodology and the equipment, the main component of the sessions was embarked upon—photo-taking and discussion. The first assignment children were set was to explore the question, ‘how do you stay healthy?’ which they investigated in the first week that they took cameras home with them. Following this initial exercise, in subsequent weeks, children were asked to explore the question, ‘what does malaria mean to you?’. The exploration of this question continued until the ninth or 10th week. Each week, images were: distributed and labelled; looked at collectively and sorted into themes; described by individuals; and discussed and analysed as a group. The final 5–6 weeks of the project were dedicated to refining themes and messages that the children wanted to disseminate to their adult caregivers using their photographs and to designing and making a range of outputs in order to do this.

All photovoice sessions were recorded using a dictaphone. In addition, throughout sessions, ethnographic notes were taken (both written notes and photographic images). Regular interviews were held with co-facilitators in order to document and evaluate the process. After photovoice sessions, transcripts of the sessions ‘proper’ and ethnographic notes were transcribed. At the end of the 15 weeks, interviews were held with participants in order to evaluate the short-term effects of photovoice.

Post-intervention questionnaires (identical to baseline questionnaires) were conducted with both intervention and control groups 3 months after the photovoice intervention in order to assess any longer-term effects on knowledge and practices relating to malaria following photovoice.

### Ethics, consent and permissions

Ethical approval was obtained from the University of Durham, UK as well as from the Research Institute for Tropical Medicine, Philippines. A research permit was obtained from the Protected Area Management Board (PAMB) of the Mantalingahan Protected Landscape (MMPL). Accordingly, Free, Prior, Informed Consent (FPIC) was obtained from all participants involved in this study as well as Municipal and *barangay* resolutions.

## Results and discussion

### Imaging malaria

During the photovoice process, participants responded to the question, ‘what does malaria mean to you?’ by photographing multiple versions of malaria. They presented selected printed images to the class and ‘visually read’ them in group discussions, providing verbal narratives that described and contextualised their images.

**Figure 1:**
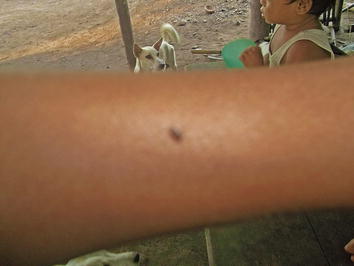
In this picture you can see a mosquito biting the skin of the hand of the child. The bite of a mosquito brings the sickness of malaria. Avoid mosquitoes landing on your hand. Hang your mosquito net every night. If a mosquito lands on you are you going to slap it? If you don’t slap the mosquito you might have the sickness of malaria.

**Figure 2:**
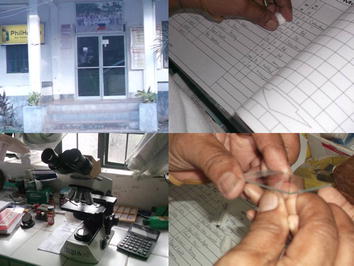
These pictures show the Health Centre and the list of the names of people who will have a check-up of their blood. We can also see the equipment that tells us if we have malaria or not. This is where we can know if our blood is positive or negative and if we are sick with malaria.

**Figure 3:**
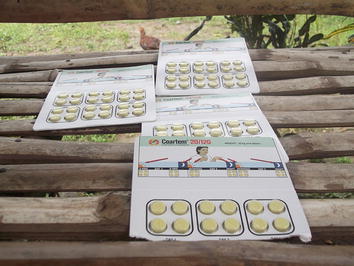
What we can see in this picture is the medicine called Coartem which is one of the primary treatments for malaria but before we take it it’s nice to consult the doctor first so that we will know if we have malaria, before we take this medicine. What I can teach people is that before we take medicine it is better to consult the Health Centre first so that we will know what kind of medicine is appropriate to our sickness.

Some of the malarias depicted align with biomedical conceptions and mirror common images of: its sources (e.g. mosquitoes); symptoms (e.g. fever); prevention practices (e.g. use of mosquito nets); diagnostic practices (e.g. use of microscopy and Rapid Diagnostic Tests) and treatment practices (e.g. use of anti-malarial drugs). This is illustrated by the examples in (Figures 1, 2, 3).

These depictions of malaria are perhaps not surprising due to the intensive malaria control and elimination activities that have gone on in Bataraza over the last decade. These initiatives are largely financed by global organisations, namely the Global Fund to Fight AIDS, Tuberculosis and Malaria who, along with local governments, fund the National Malaria Control Programme (NMCP). The NMCP is decentralized and delivered locally through Rural Health Units and *Barangay* Health stations. The international, national and local agencies responsible for malaria control largely define malaria within the biomedical framework and have pushed forward a number of community education activities to disseminate this conception of malaria as well as various technical solutions in an effort to tackle it.

**Figure 4:**
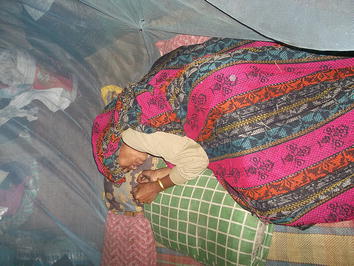
In this picture you can see the feelings of the people—chilling and headache and feeling cold. I took this picture of my mother in my house. This is what people feel when they have malaria. I can teach people that they should take care to rest after work so that they do not get sick with malaria.

**Figure 5:**
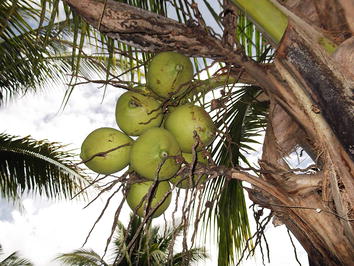
Coconuts are very good for your health because they make you fat and strong. Eat coconuts and plenty of rice if you want to avoid getting sicknesses like malaria.

**Figure 6:**
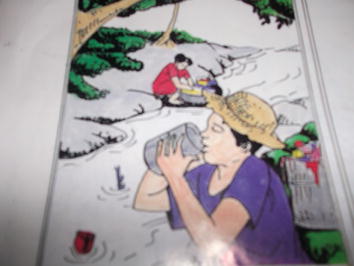
Shown in this picture is a man drinking dirty water directly from the river. It’s not good for health because it carries the malaria disease. I can teach the people to avoid drinking dirty water directly from the river. It carries malaria disease and I can tell the young people don’t drink dirty water from the river. Maintain clean drinking water to avoid malaria.

**Figure 7:**
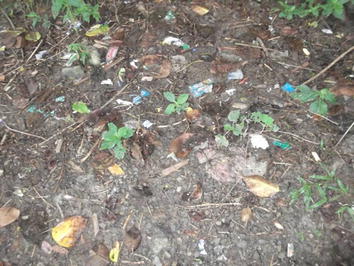
In the picture we see it is full of garbage. There are a lot of plastics. The different kinds of plastic and garbage bring sickness to the people like malaria. They say if you pick up garbage you can get malaria.

However, in addition to these depictions, participants also photographed malarias that aligned with more local understanding of the body, health and well-being, which are often neglected by health practitioners. In the case of the Palawan, these neglected malarias are structured around the central tenet of balance. This is illustrated by Figures 4, 5, 6, 7.

Multiple studies that explore health in various parts of the Philippines [11, 12] or amongst migrant Filipino groups have identified the principle of balance (*Timbang* in Tagalog) as central to concepts of health, nature and social relationships [13–15]. As Anderson [15] articulates, ‘health is a result of balance, illness is usually the result of some imbalance’ (ibid.: 815) and the basic logic of health and illness involves both prevention (avoidance of inappropriate behaviour that causes imbalance) and curing (by restoring balance). This is exemplified by the fact that the Palawan do not have a specific term for “health” or “healthy” but associated terms include *metaba* (Palawano for fat or chubby) and *masubug* and *kaya* (Palawano for strong). People therefore feel they are well when they are strong, able to work or play and have a healthy appetite. People speak of fatness as a positive indicator of well-being and thinness as an indicator of illness. Conversely, people know they are sick when they feel weak or are unable to get up, work, play or eat. It is at this point that people will seek treatment. Pain is also an indicator of ill-health the Tagalog word *sakit* literally translates into pain, and is used in several Austronesian languages as synonymous with ‘illness’ [16].

In terms of malaria more specifically, the vast majority of participants were unable to give clear answers regarding *what* exactly malaria is beyond it being a sickness (*sakit*). The most common definition given is that malaria is disequilibrium inside the body although it is rarely expressed in exactly those terms. Instead, people refer to an internal bodily state: it *is* too much heat, too much cold, too much dampness inside the body etc. Similarly, the majority of people cited one or a list of manifestations of that state (what biomedicine refers to as symptoms): it *is* dizziness, headache, fever, chilling, stomach ache, vomiting etc. Interestingly, imbalance is usually expressed as an excess rather than deficit of something.

In most cases, internal imbalance is caused because of the body’s interaction with external elements, which correspond the earth, water, fire and wind. Central to this idea, is the congruence between the internal body and the external environment. A commonly cited cause of malaria is *pasma* which loosely translates as ‘exposure illness’ [17]. This tends to refer to the application of cold to heat or vice versa or applying more heat to existing heat. The sun is the primary cause of too much heat in the body and ‘too much sunlight’ is a commonly cited cause of malaria. The hot and cold dichotomy is the most significant in relation to health but participants also referred to the dichotomous elements of wetness and dryness which is absent from many parts of the world where hot/cold complexes exist [18]. These two elements also need to be kept in harmony and are particularly affected by sudden changes in weather, especially fluctuations between dry sunlight and wet rain and vice versa. These fluctuations also cause malaria to ‘come out’.

As well as natural external elements like the weather, practices that people engage in also change the balance of internal bodily states. Physical activity and exercise are heat generating activities and partaking in too much physical exercise or work can aggravate excessive or unnatural heat in the body which can lead to illness as illustrated by the example (Figure 4) above of the woman suffering from fever and chilling as result of not taking sufficient rest after work.

Food is another important factor which affects balance in the body. Warmth is generally associated with health making food necessary for maintaining warmth, strength and fatness. It is very common for people to cite hunger as a cause of their illness and is generally associated with the body becoming too cold. While participants associated many foods including vegetables with being healthy, it is important to note that not all foods will necessarily satisfy hunger. Rice is at the centre of every Palawan (and Filipino) meal and is a huge part of Palawan social and spiritual life. In terms of health more specifically, people reported the significance of rice in terms of satisfying hunger. In fact, a meal is not really considered a meal unless it contains rice (and often rice alone is enough) precisely because no other food can fill you up in the same way. This is illustrated by the example (Figure 5) given above of the coconuts.

As well as imbalance of elements, people also describe imbalance in the body that is caused by the introduction of an external agent, specifically into the blood, but they are less clear about what the agent itself is. As one child described it, ‘something scatters inside you’. When people do specify what the agent was, it is most commonly described as ‘dirt’ but the principal of imbalance is the same; ‘there is too much dirt inside you’. I found that amongst patients there was no reference to pathogens and none of the young people or adults that I spoke to within the Palawan community referred to the viruses, bacteria or parasites. In terms of how external agents like dirt enter the body, mosquitoes were the most commonly cited explanation given in interviews. Participants expressed that mosquitoes can introduce dirt into the body through a variety of methods. Firstly, mosquitoes are attracted to and live in dirty places and to lay their eggs in stagnant and dirty water. After being in such dirty places, mosquitoes have the potential to introduce dirt into the body directly by biting people. Secondly, directly drinking ‘dirty water’, eating ‘dirty food’ or breathing in breathing in ‘dirty air’ contaminated with mosquito eggs or saliva was another possible source of getting malaria. Thirdly, some young people also mentioned that swimming in dirty water contaminated with mosquito larvae was another possible way to get malaria. As well as through mosquitoes, external agents like dirt can also be introduced into the body through the consumption of dirty water and food as the example (Figure 6) above of the man drinking dirty water from the river reveals. However, as the last example given above illustrates, malaria can also be caused through direct contact with dirty things like rubbish which contaminates hands or bodies (Figure 7).

**Figure 8:**
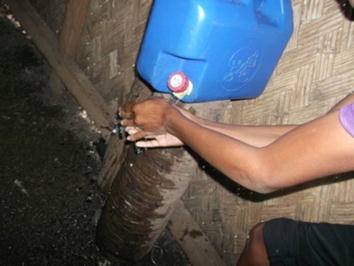
This is a picture of my classmates washing their hands. You can see a water jug, water and the wall. That’s me. This is to show that the hands are clean because of washing hands. It’s good so that there is no dirt in your nails. You will get a lot of sickness from the dirt in your nails like stomach pain, headache and malaria.

**Figure 9:**
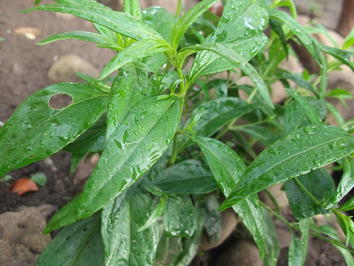
In this picture you can see pito–pito. This is an effective medicine for malaria and it is planted beside our house. When we are sick, we boil pito–pito and then put it in the glass and drink it and the taste is acrid. We can teach the people that every family must plant pito-pito.

**Figure 10:**
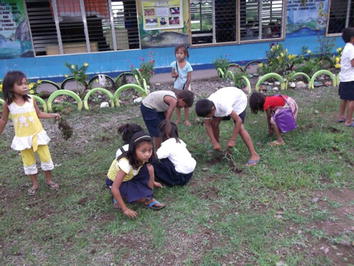
In this picture you can see dirty surroundings. We need to clean the dirty surroundings and remove the weeds so that we can avoid the sickness of malaria.

Consistent with these malarias that are brought about by imbalance, participants photographed themselves and members of their family and community engaging in a number of practices which are orientated towards restoring and maintaining balance. This is illustrated in the examples given below (Figures 8, 9, 10). As with causes of different versions malaria, these socially embedded practices are often ignored by health professionals who consider behaviours that not fit the biomedical view to be at best, irrelevant or incorrect and at worst, responsible for ‘delayed’ seeking of ‘proper’ treatment.

### Altering malaria knowledge and practice

As well being an effective means to illuminate multiple malarias and the practices that surround them, photovoice also enabled participants to learn new things and significantly, teach these things to others using their images. By the end of the project, the majority of young people certainly felt that their engagement and the outputs they produced enabled them to communicate what they knew about health and malaria with their family members.

Although participants initially found the prospect of teaching others difficult, by the end, they all felt that this was something they were now much better at:

‘At first I was shy but now I am able to teach the people about malaria’

(Female photovoice participant age 12).

‘Before, I cannot do any kind of announcements but right now I am able to make announcements. Like, if I had a picture of a mosquito then now I can explain that to other people’

(Male photovoice participant age 14).

The majority of participants expressed this ability to teach in terms of feeling less ‘shy’. This is particularly significant in the context of Palawan culture as shyness is a normative and even valued personal trait, especially for young girls. As Macdonald [19] explains *metemen* (Palawano for meekness and mild manners) is ‘valued to a high degree, contrary to assertiveness and arrogance (*dakag*), which are the epitome of offensive behaviour’ (ibid.: 139). This is coupled with the relational way in which identity is created which means that older members of the family (particularly men), are not always likely to heed to the advice of young people, nor consult them in decision-making processes including those related to health. Here, photovoice enabled young people to have the confidence as well as the *means* to communicate what they had learnt with older members of their family including their parents, and more significantly perhaps, enabled parents to listen and learn. In the context created by the photovoice project, this was considered both appropriate and positive.

The largely positive change in young people’s ability to communicate with others was reflected in questionnaire results as there was a statistically significant increase in the number of adults from the intervention group that had heard information about malaria from young people compared to the control group (Table 1).

**Table 1:** Table showing sources of malaria information that adults reported receiving

Sources of information on malaria	Intervention			Control		
	Before *n* (%)	After *n* (%)	*p* value	Before *n* (%)	After *n* (%)	p value
Radio	29 (82.9)	26 (83.9)	0.912	30 (62.5)	32 (72.7)	0.296
TV	17 (48.6)	15 (48.4)	0.988	23 (47.9)	10 (22.7)	0.012*
Poster	16 (45.7)	15 (48.4)	0.828	21 (43.8)	12 (27.3)	0.100
School	23 (65.7)	21 (67.7)	0.862	33 (68.8)	26 (59.1)	0.335
Teachers	23 (65.7)	21 (67.7)	0.862	33 (68.8)	28 (63.6)	0.604
Adults	14 (40.0)	18 (58.1)	0.143	31 (64.6)	18 (40.9)	0.023*
Young people	*8 (22.9)*	*15 (48.4)*	0.030*	24 (50.0)	15 (34.1)	0.123
Health workers	29 (82.9)	27 (87.1)	0.632	42 (87.5)	37 (84.1)	0.639

The italics refer to the significance of this result (also indicated by the *).

More significantly, both young people and parents expressed that as a result of their children’s teaching, they were able to learn things that they considered to be ‘new’ or to do things ‘differently’. Again this was something that was welcomed and the favourable reaction that parents in particular had to taking direction from young people seemed to be because of the unique circumstances and context created by photovoice.

The idea that parents were exposed to ‘new’ teachings was perhaps surprising as, I myself had witnessed many of the same messages being taught in other health related campaigns initiated by the Rural Health Unit (RHU) and National Malaria Control Programme (NMCP) and some of the practices that young people were advising others to do (and that participants reported they now did as result) were things that I had observed, and participants had reported in interviews and questionnaires, doing already in relation to malaria like sleeping under mosquito nets and cleaning their surroundings.

‘I told them we need to use a mosquito net every night in order to avoid mosquito bites. Even my aunties told me ‘thank you for informing us and giving us this new information so now we will obey you and sleep under the net’. I also told them ‘oh aunty you know paracetamol and amoxicillin is not a medicine for malaria’. I also told my parents. I also told them to avoid mosquito bites to avoid getting malaria’

(Female photovoice participant age 12).

‘I also told my neighbours that if you sleep in the night then use a mosquito net. I told the very old ones. They said ‘ah ok’. They didn’t know that before’

(Male photovoice participant age 10).

‘I told my mother. I told her to clean up the surroundings so that there is no place for mosquitoes to live and there is no-where for them to lay their eggs… She said ‘ok, let’s go clean up together then. Now I clean up the surroundings… This helped the whole family especially in my family because we are cleaning up the surroundings and sleeping under the mosquito nets now so it really helped us’

(Male photovoice participant age 12).

‘I told my mother. I said ‘mother we need to use a mosquito net regularly’. She said ‘ok from now on I will use a mosquito net every night’. Yes we are doing that now… Also we need to plant herbal medicines. I told to my father. He said ‘ok I will plant herbal medicines’. But I didn’t see him do that yet’

(Female photovoice participant age 11).

‘Yes. I told my older sister and my mother to clean up the surroundings every day. They said ‘Yea you are right; we will clean the surroundings so we can avoid malaria’. I also told my aunty [that] paracetamol is not for malaria. These were new things for them also’

(Female photovoice participant age 12).

This disjuncture could be explained by many possible reasons. As described above, malaria is used as a blanket term to describe a wide spectrum of illnesses that result from imbalance and as such, an equally wide spectrum of beliefs, knowledge and practices surround it. In light of this, what was considered to be ‘new’ or ‘different’ varied considerably between individual participants. This is coupled with the fact that the official awareness campaigns delivered by the RHU and NMCP, combine information on many febrile illness like malaria, dengue, Urinary Tract Infections and Sexually Transmitted Diseases which are presented to audiences in one ‘lecture’ style session. Many participants found these sessions confusing and as a result, were not clear on the distinctions between malaria and other illnesses nor did they always necessarily associate the objects and practices surrounding illnesses in general with any one ‘disease’. As such, some participants told me that even though they used mosquito nets to prevent mosquito bites, they associated this practice with many illnesses, malaria being just one.

However, there are other reasons why participants may have considered the teachings from photovoice more compelling and therefore ‘new’ when compared to other kinds that concern the methodology itself. The nature of the knowledge that was being generated by young people and then passed on to others was itself considered by participants to be particularly engaging due to its visual nature. In evaluation interviews, young people expressed that it was the pictures directly that compelled them and others to learn and teach things. They felt this power of photographs was not something they had necessarily given much thought to before doing the project. As these quotations demonstrate:

‘It’s better when there are pictures because you alone can read the pictures and get their meaning’

(Female photovoice participant age 11).

‘[Pictures help you learn] because you can see the message in the picture… When you speak with a picture it is easier than when you speak without picture. Because it helps you to remember, even things from the past’

(Female photovoice participant age 15).

*‘*Researcher: *Do you think using pictures helped you to learn new things?*
Participant: *Oh yes*
Researcher: *Why do pictures help you to learn?*
Participant: *When people are looking at the pictures they are happy. The pictures also help to prove the reality*—*they provide an example*
Researcher: *Did you think that before?*
Participant: *No, only now we realised that pictures are so important’*
  (Female photovoice participant age 12)

As well as being a compelling way to both teach and learn due to the visual nature of the information, the images produced in photovoice were also considered by participants to be authentic and directly relevant to their lives because they were generated by the participants themselves. As such, they produced produce something familiar to audiences with ‘real’ faces and places—recognisable participants, parents, friends and neighbours enacting their lives in homes, gardens and the wider community. Images *showed* what audiences described as ‘reality’.

However, these largely positive outcomes were not uniform across all participants as some young people reported that they had not communicated what they had learnt with others. Some young people reported that even though they did feel less shy or had learnt new things as a result of the project, they still did not feel able to teach others new things. Furthermore, although the majority of young people reported positive outcomes, this does not necessarily mean all participants had the same experience or derived the same benefits. Young people are, after all, not homogenous groups, with similarities or connectedness determined simply by their similar age [20]. This highlights an important point about participatory research with young people but also research more generally: that the views voiced by some children in research, cannot represent all children everywhere (ibid.).

Similarly, some adults reported that their children did not talk to them much about the project or that they did not see the outputs that they produced. This is reflected in the questionnaire data in which less than half of adult caregivers from the intervention group reported that had heard information about malaria from young people compared to the control group (Table 1). In some cases this was due to practical reasons as for the father below but in others, parents ‘forgot’ what their children had told them as in the case of the mother below:

‘[My son] talked to his mother about things because many times I am away working… I did not go to the event because I was at work’

(Father of male photovoice participant).

*‘*Researcher: *Did* [your daughter] *talk about the project?*
Participant: *Sometimes she is telling me about it*
Researcher: *Did she tell you about any of the things she learnt?*
Participant: *Some of the things but I forgot already what’*
  (Mother of female photovoice participant)

One of the primary aims of this project was to assess whether doing photovoice could not only illuminate practice but also alter how people *did* [21] malaria. The nature of the photovoice method means that what each participant contributed to the project was different; reflecting their own lived experience, knowledge and expertise. Similarly, what they then got out of the project (and thus communicated to others) was equally varied. In part, this was is due to individual experience but also because within this study, photovoice was conducted in five different groups and each group project as a whole, took on a different course. Consequently, although all young people reported that they had learnt something new about malaria, what this was, varied between groups and even individuals.

For example:

‘Before, I thought that malaria came from dirty water but I made a mistake here because you can get malaria only from the bite of a mosquito. And also like herbal medicines, I thought before that they are a treatment for malaria but I am wrong’

(Female photovoice participant age 11).

‘Yes, I learnt that is important to sleep under a mosquito net. Before, I did not have deeper knowledge of that before. I knew something about it but not a lot’

(Male photovoice participant age 14).

‘I also learnt about throwing away trash. I was doing it before but I didn’t know why’

(Male photovoice participant age 13).

‘Yes—I learnt you should have a lot of animals in the house—to take care of a lot of animals so that mosquitoes will bite them and not the people’

(Female photovoice participant age 12).

‘I learnt mosquitoes bite people and then you can get sick’

(Male photovoice participant age 11).

‘I learnt that garbage is where mosquitoes lay their eggs’

(Male photovoice participant age 12).

‘Yes—to eat vegetables. Vegetables make you fat’

(Male photovoice participant age 10).

As well as changes to knowledge, the majority of young people reported that, as a result of photovoice, they engaged in a number of new practices or that they carried out existing practices in different ways, for example more regularly. As with knowledge, there was variation between groups and individuals regarding which practices were altered and how:

‘Now I clean up the surroundings, make smoke regularly and know to allow the spray man to spray the house’

(Female photovoice participant age 12).

‘Since your project, I noticed some changes. I have seen her cleaning up the surroundings. If there is some stagnant water, she fixed the canal so that the water will flow’

(Mother of female participant).

‘I learnt to use regularly the mosquito net. I wasn’t doing that before. Before, my parents were using the mosquito net but not me. Now I am using it every night. I didn’t explain to my parents, I just did it’

(Male photovoice participant age 12).

‘From the time he was involved in the project, he is helpful now’

(Mother of male photovoice participant).

‘Dirty water, I throw it away so there is no place for mosquitoes to lay their eggs. [I did that] just now, not before’

(Male photovoice participant age 12).

In addition, many young people reported that their actions in communicating with their family members, friends and neighbours resulted in these people even changing some of their practices but again, what specifically altered varied between individuals.

‘Now we are using a mosquito net. Before we are not using them. We were only using them sometimes but now we use them regularly. The whole family is now using them’

(Female photovoice participant age 12).

‘I told my mother. I told her to clean up the surroundings so that there is no place for mosquitoes to live and there is no-where for them to lay their eggs… She said ‘ok, let’s go clean up together then’

(Male photovoice participant age 12).

These changes were also reported by some of the adults that I spoke to:

‘We did number 6 [from the checklist] together—burn smoke always so that mosquitoes go away. Also throwing away dirty water and using a mosquito net’

(Mother of female photovoice participant).

‘Yes, [I learnt] you need to clean up the surroundings like the garbage and the trash so that you can avoid the sickness of malaria. Because mosquitoes lay their eggs in the dirty trash so we need to burn them. Not just the trash but the places like that [points] we need to clean it up so that there is nowhere for mosquitoes to live. I have heard that from that programme you have there and I also I saw that in the pictures. Also [my son] showed us his pictures and I saw pictures of dirty water so I know we need to clear dirty water to avoid mosquitoes having a place to live… We have been doing those things together like sleeping under a net and cleaning up our surroundings. [My son] is the one who is helping me so much now’

(Mother of male photovoice participant).

## Conclusion

This article has shown that photovoice is an effective method for re-imaging malaria. It allowed participants to *depict* and *describe* multiple forms of malaria and the practices that they engage in *in context.* Some of these versions of malaria are often silenced, ignored and even neglected [2] by official control programmes. Photographs confer many advantages in the research process and can be a rich source of data about lived experience. As such, photovoice presents a useful means of carrying out interpretive phenomenological research [22] as it allows participants and researchers to access the lived experience of practice in a way that is meaningful to participants rather than that which is most relevant and meaningful to researchers. However, photographs, like all other objects, move through time and space and as they do so, their meanings and significance change as they acquire social lives [23]. In the photovoice process, photographs are created, that *depict* one version of reality captured because of the intentionality of the photographer at one moment in time. However, they then move throughout the research setting between the photographer, audience, participants and researchers and wider community as they acquire various narratives, meanings and uses. As such, photovoice does not so much ‘uncover’ reality but *depict* different, contextually specific versions of it as they are *done*. This partiality is exacerbated in the photovoice process because the skills needed to conduct it such as ‘visual literacy’ to ‘read’ images and ‘verbal literacy’ to contextualise them are not universally homogenous but skills that can develop over time and in different culturally mediated ways throughout the whole process. Furthermore, the fact that the method relies on group discussion runs the risk that some voices may be elevated whilst others are silenced.

Beyond simply re-imaging or depicting multiple versions of malaria, photovoice also had a potentially transformative effect, in line with PAR more generally. Photovoice acted as a means for participants and researchers to: visually *depict* everyday practices; collectively gain a deeper understanding of this doing; and then seek ways in which to make changes *in line* with this joint understanding. As such, *doing* photovoice went some way to helping to re-imagine malaria by providing an effective means for re-imaging and re-doing it differently.

## Abbreviations

NMCP: National Malaria Control Programme
PV: photovoice
PAR: participatory action research
RHU: rural health unit
